# Glaucoma among the Malaysian Community

**DOI:** 10.1155/2020/4859496

**Published:** 2020-08-01

**Authors:** Redhwan Ahmed Al-Naggar, Hisham Alshaikhli, R. R. Al-Rashidi, Bahaa Saleh

**Affiliations:** ^1^Community Medicine Department, Faculty of Medicine, UKM, Malaysia; ^2^School of Public Health and Interdisciplinary Studies, Faculty of Health and Environmental Sciences, Auckland University of Technology, Auckland, New Zealand; ^3^Department of Dentistry, Al-Kut University College, Wasit, Iraq; ^4^School of Science and Engineering, University of Waikato, Hamilton, New Zealand

## Abstract

**Background:**

According to the WHO, glaucoma is the second leading cause of blindness worldwide. About 50% of the world's glaucoma cases come from the Asian population, and in Malaysia itself, the prevalence of glaucoma is increasing. However, glaucoma is still a foreign word to our community despite the high prevalence. Therefore, there is an urgent need to determine the awareness of glaucoma among the community and its associated factors.

**Results:**

This study showed that only 25.2% of our respondents were aware of glaucoma and it is associated with ethnicity, religion, education, and household income. Besides, among those who were aware, they fall into the group of poor knowledge of glaucoma. On the other hand, the knowledge of glaucoma was associated with occupation and the awareness of glaucoma by definition. The validated questionnaire was distributed and the data were analyzed by SPSS software using *t*-test, one-way ANOVA, and chi-square test.

**Conclusion:**

Awareness and knowledge of glaucoma in this population is low. These findings suggest that there is a need for an efficient information and education strategy to be designed and conducted to increase the awareness and knowledge of glaucoma so that early detection can be made and effective management of individuals with this condition can be delivered.

## 1. Introduction

Glaucoma is defined as a group of ocular disorders that involves progressively damaged optic nerve and characterized by peripheral loss of vision [1]. It can be contributed by many factors such as age, family history, race, gender, and other comorbidities such as diabetes and hypertension [2].

WHO has reported that glaucoma is the second leading cause of blindness worldwide [3]. The increasing trend of prevalence of glaucoma has become a worrisome issue in Malaysia especially when 50% of the world's glaucoma cases come from the Asian population [4].

Glaucoma is still a public health problem also in developing counties, and in Finland, glaucoma is the second cause of permanent visual impairment among elderly persons [5]. However, Europe has the lowest prevalence of glaucoma at 2.93% in people over 40 years old [6]. In Ireland, the prevalence of glaucoma was 2.83% among the population aged 50 years and older [7].

Every year, World Glaucoma Week (WGW) takes place for one week during March. The objective is to promote awareness about glaucoma. This is an opportunity to conduct such an event to discuss how glaucoma affects vision and how the lives of patients and their families are affected [8].

The major consequence of glaucoma is bilateral blindness. This leads to impairment in quality of life by the disability to do visual tasks, reduce mobility, and become more dependent [9, 10]. Visual impairments in glaucoma patients usually lead to a higher risk of getting depression and more prone to get personal injuries such as motor vehicle accidents especially in the older age groups [11–13].

As the physicians and community are usually concern about the major health issues such as cardiovascular disease, we often forget about the non-life-threatening conditions yet debilitating such as glaucoma itself. This can be shown by a lack of awareness and knowledge of glaucoma in some communities especially in low-income countries and low educational status communities [14]. This has been shown by a study done in India which revealed a 2.8% awareness and knowledge about glaucoma [15].

It is very important to instill the awareness and knowledge of glaucoma to the community as it is a treatable [12]. Glaucoma usually presented with minimal or no manifestations and it is usually very difficult to have early detection of glaucoma. Hence, with knowledge and awareness of the community, early detection can be made and effective management can be delivered to prevent or delay the progression of glaucoma [4]. Besides that, millions of money can be saved by preventing glaucoma, as it requires lifelong therapy which is costly to the family and the government [16, 17].

A study showed that risk factors for open-angle glaucoma include increased age, ethnicity, family history of glaucoma, increased IOP, myopia, and decreased corneal thickness [18]. Some other possible risk factors for glaucoma are male gender, diabetes, hypertension, eye injury or surgery, history of steroid use, migraine headaches, and sleep-related breathing disorders.

Studies from India revealed the associated factors of awareness and knowledge of glaucoma which include age, gender, marital status, educational level, socioeconomic status, religion, diabetes status, and family history of glaucoma. All the studies showed that respondents who have high educational status, high socioeconomic status, diabetes, and family history of glaucoma have high awareness about glaucoma [14, 17, 19–22]. The main source of information in India regarding awareness of glaucoma was physician, followed by media and family history [22]. Studies from University Putra Malaysia indicated that people living and working in Malaysia rely more on television to access relevant information [23]. High exposure to mass communication media such as television, radio, and magazines has created a greater awareness among the population [14]. Sufficient access and proper usage of ophthalmic care services can create greater awareness and exposure to information about the various eye diseases including glaucoma.

An ocular examination done during the health education program may help in the early detection of glaucoma among those who are unaware or not willing to seek examination and treatment [14]. Examinations include vision tests, optic nerve evaluation, eye pressure measurement, a test of the eye's drainage angle, and visual field assessment of each eye. Information from these examinations is compared at regular intervals to determine glaucoma progression damage [24].

In Malaysia, there is a lack of studies regarding awareness and knowledge of glaucoma. A study had been done in University Malaya revealed that 71.5% of respondents had awareness regarding glaucoma. However, this study was conducted in a very specific population which is the university staff only [25]. Therefore, the objective of this study is to determine the knowledge and associated factors of glaucoma among the community.

## 2. Methodology

This study is a cross-sectional study that aims at determining the determinants of awareness and knowledge of glaucoma in the community over a while. The variables in this study include demographic details of the community (age, gender, race, religion, educational status, occupation, household income, and family history of glaucoma) and awareness and knowledge of the community on glaucoma. This study was conducted at Shah Alam, Selangor, Malaysia. The estimated population who lives in that area is about 1455 people with a total of 237 units of houses. The sample size of this study was 329, and the sample size was calculated by using Epi Info 7 based on the population size (1455), expected frequency (50%), confidence limit (5%), design effect (1.0), 95% confidence level, and cluster (1). The inclusion criteria for this study were Malaysian residents, able to understand Bahasa Melayu or English, aged above 18 years old, and willing to participate in the study. The data collection tool used in this study is an adopted questionnaire regarding awareness and knowledge on glaucoma [15, 21]. The questionnaire was developed in stages which included literature search, discussion, and pretesting the questionnaire to ensure good content validity. Then, a pilot study was done among 30 participants before the actual study was initiated to pretest/validate the set of questions in the questionnaire. The result of the pilot study has helped us in getting a clearer idea of what we wanted to know and helped in refining our research hypothesis.

The questionnaire consists of three sections with a total of 26 questions. The first section was about sociodemographic characteristics which included nine questions. The second section was about awareness of glaucoma. This section contains four questions which aim to determine the awareness of the respondent on glaucoma. The first two questions must be at least one with the answer “Yes” to consider the respondent as aware of glaucoma. If the respondent is unaware, they need not proceed to the other question. This domain also contains questions that elicit the individual source of information regarding glaucoma. The third section was about knowledge of glaucoma. This third section comprises of 13 questions that assess the respondent's knowledge of glaucoma. There are 3 answer options, which are “Yes,” “No,” and “Not sure.” For each correct answer, 3 marks will be credited, if “Not sure” will be given 2 marks, and each wrong answer will be given 1 mark. The total score is 39 marks if all questions are answered correctly. The categories of knowledge scores were decided by using an arbitrary scoring system.

Sampling method used in this study was simple random sampling technique. This technique is one of the probability sampling techniques in which each eligible candidate has an equal chance to be selected to answer the questionnaire set. Firstly, we prepare a sampling frame which is the list of eligible candidate names. By using Research Randomiser Software, 334 names were selected and each name that corresponds to the chosen number was interviewed by the researchers. Ethical approval was obtained from the Research Ethics Committee of the Research Management Institute of Universiti Teknologi MARA, Malaysia. For data analysis, the data were entered, cleaned, and analyzed by using SPSS software. Appropriate statistical tests such as *t*-test, ANOVA test, and chi-squared test were used according to the type of variables, and the significance level will be taken at 95% or a *P* value of less than 0.05.

## 3. Result

A total of 329 respondents answered the questionnaires completely. The mean age of the participants was 34.94 ± 10.608. Two hundred and thirty-six (71.7%) residents were Malay. Two hundred and fifteen (65.3%) of the respondent had education up to the secondary education level. Two hundred and thirty (69.9%) respondents are employed. The mean household income was 550 USD. Most of the respondents (88.1%) did not have chronic illnesses. From thirty-nine (11.9%) respondents who had chronic illness, most of them had hypertension (69.2%). Seventy-five (90.4%) respondents did not have family members with glaucoma (Table 1).

For the awareness, 63 (19.1%) of the respondents have heard of the term glaucoma and 50 (15.2%) of them have heard of the definition of glaucoma. A total of eighty-three (25.2%) respondents were aware of glaucoma. For the sources of information about glaucoma, 37 (44.6%) of the respondents heard about glaucoma from television, followed by 31 (37.3) who had heard of glaucoma from newspaper and magazine (Table 2).

Respondents that had answered correctly that anyone can have glaucoma were twenty-six (31.3%) respondents. Most of the respondents (71.1%) had answered correctly that glaucoma is treatable. Thirty-three (39.8%) respondents know that glaucoma is not the same as cataract. Fifty-seven (68.7%) respondents answered correctly that an eye doctor's check-up is the only way of early identifying glaucoma. Fifty-three (63.9%) respondents know that glaucoma is not an infectious disease. Only eighteen (21.7%) respondents know glaucoma is not caused by reading books, newspapers, or using a computer for a long period. Thirty (36.1%) respondents answered not sure to glaucoma runs in families. Only twenty-seven (32.5%) respondents answered correctly that people aged 40 years old or above are the ones most likely to have glaucoma. Thirty-three (39.8%) respondents know that glaucoma has an asymptomatic course. Out of 83 respondents, only twelve (14.5%) respondents know in glaucoma, vision is not affected in the early course and only sixteen (19.3%) respondents know glaucoma affects the side vision. Fifty-six (67.5%) respondents answered correctly that glaucoma develops slowly over time. Most of the respondents (66.3%) know that glaucoma causes blindness (Table 3).

The sociodemographic factors associated with awareness of glaucoma in the community including gender, race, religion, occupational status, educational level, history of diabetes mellitus, hypertension, heart diseases, and hypercholesterolemia. Three factors are statistically significantly associated with awareness of glaucoma which are race (OR = 5.720), religion (OR = 5.836), and educational level (OR = 4.180) (Table 4).

As for the associated factors, the respondents who have a higher mean of household income are aware of glaucoma compare to those who are not. Furthermore, the respondents who are aware of the definition of glaucoma have a higher mean of knowledge (Table 5).

## 4. Discussion

The overall awareness in this study is low; 25.2% were aware of glaucoma, 10.03% are only aware of the term “glaucoma,” 6.08% are aware by the definition of glaucoma, and a total of 74.77% are not aware of glaucoma. Similar findings reported by a study done in Toronto, Canada, reported that glaucoma knowledge in the community is inadequate [21]. In contrast to our study, a Malaysian study showed that 71.5% of respondents had awareness regarding glaucoma. However, this study was conducted among the university staff only; hence, a direct comparison may not be applicable [15]. The awareness level is higher compared to developing nations like India (0.27%), Nepal (2.4%), and Ethiopia (2.4%), but low compared to reports from the United States of America (72%–79%) [11, 12].

For the sources of information about glaucoma, 44.6% used television as the main source of information on glaucoma followed by newspapers and magazines (37.3%), relatives and friends (24%), radio (10%), and newspaper/magazine (10%). This study is parallel to a study conducted in University Putra Malaysia which indicated that people living and working in Malaysia rely more on television to access relevant information [23]. Among the urban community, they have high exposure to mass communication media, thus leading to greater awareness of glaucoma [14]. The fact that television is a powerful and exciting means of information dissemination, due to its convenience and flexibility, wide coverage of target groups, and its ability to deliver a strong impact, must be utilized by healthcare professionals in promoting glaucoma awareness and knowledge, for example, increasing glaucoma-related advertisements and discussions on glaucoma on talk shows or health-related TV shows. At the same time, it is important that information and knowledge dissemination via family and friends are being done in the public. In a German survey, the main source of information reported among those who are aware of glaucoma is friends and relatives [26]. Another study in India reported that mass media was the main source of information on glaucoma [27].

Our study showed that there is a significant association between ethnicity and the level of awareness of glaucoma, in which Malays showed the highest association compared to other races. The significant association between ethnicity and awareness of glaucoma in our study may be contributed to the high number of respondents from the Malay ethnicity. Comparing to the previous study, according to a survey done in Florida, USA, ethnicity was significantly associated with awareness of glaucoma. They reported that African American race and Hispanic ethnicity increased the likelihood of being unaware of glaucoma [23]. Studies from the United States reported that black people have a higher prevalence than whites [28, 29].

Previous studies in the United States showed that the prevalence of glaucoma is 4 to 5 times higher among folks of African descent than in those of European descent [30, 31]. Furthermore, several studies reported that there are more visual impairment and faster progression of glaucoma in folks of African descent than in other ethnicities [31, 32]. This may due to that the healthy individuals of African descent have, on average, thinner corneas and greater optic disc and neuroretinal rim area, larger cups, larger cup-to-disc ratio measurements, and thicker retinal nerve fiber layer than others [33, 34]. In contrast, one study reported that ethnicity was not significantly associated with awareness of glaucoma [35].

This study showed a significant association between religion with the level of awareness of glaucoma. In contrast to our findings, a study done in Ethiopia showed that awareness of glaucoma in relation to religion was not statistically significant [36]. However, a study done among Indians revealed that Hindus were 4 times more likely to be aware of glaucoma when compared to Muslims [35].

Educational level has a significant association with the awareness of glaucoma in this study. Respondents that have tertiary education have a higher proportion of glaucoma awareness (49.3%) compared to those who do not have tertiary education (18.8%). Similar findings reported by a study conducted in Australia showed that knowledge of glaucoma was related to educational level and knowledge of other eye diseases [37]. To the best of our knowledge, there is no study conducted in Malaysia to investigate the association between educational level and glaucoma awareness. However, many studies conducted in developed and developing countries reported that educational level has a significant association with an awareness of glaucoma [14, 15, 22, 36–38].

The household income showed a significant association with glaucoma awareness. Respondents that are aware of glaucoma had a higher mean of household income compared to respondents that are not aware of glaucoma. Our finding was consistent with a study conducted in India, in which they found that awareness of glaucoma was significantly higher in the high socioeconomic group [14]. Our findings also similar to a study done in Toronto revealed that poverty was associated with lower levels of glaucoma knowledge [21].

There was no association between age, gender, occupational status, and family history of glaucoma with glaucoma awareness. These findings are consistent with the studies conducted in India [14]. Regarding the age, studies conducted in India and Australia reported that older respondents had a high level of awareness compared to the young age group and there are also studies reported that being female had a higher level of glaucoma awareness while some other studies reported that male has a higher level of glaucoma awareness [16, 20, 25]. Another study reported that skilled workers had a high level of glaucoma awareness compared to other types of occupations [20]. The previous studies also showed that those who have a family history of eye diseases or a family history of glaucoma were significantly more aware of glaucoma [22, 25, 37]. Prabhu and others stated that those who have a history of diabetes have more awareness about glaucoma [22].

From our study, we found that occupational status is significantly associated with the level of knowledge in the community. From our study, we found that occupational status is significantly associated with the level of knowledge in the community. Working respondents have a higher knowledge of glaucoma compared to those who are not working. This is consistent with the study conducted in Australia in which they found that knowledge of glaucoma increased higher occupational prestige [28]. In contrast to our findings, few studies revealed there is no association of occupational status with knowledge of glaucoma [39].

There was no association between age, gender, ethnicity, educational level, occupational status, household income, chronic illness, and family history of glaucoma with knowledge of glaucoma. A study conducted in University Malaya revealed that females, older people, and those having a family history of eye diseases were significantly more knowledgeable about eye diseases [25]. The differences of the findings of these studies may be due to the target population, our study was conducted among the general population, and however, the other study was conducted among university staff in which they are highly educated.

The strength of this study is that it is the first study on glaucoma awareness and knowledge among the public in Malaysia and it is also the first study that determines the association between awareness of glaucoma by term or definition with knowledge of glaucoma. The findings of this study provide a baseline on awareness and knowledge of glaucoma among the community in Malaysia. Thus, this study implication can be used for future planning of early detection and management of glaucoma. However, there were some limitations in our study. One of them was our respondents were limited to a small study population, and thus, it does not represent Malaysia as a whole. Besides, our study did not provide the prevalence of glaucoma in Malaysia, and so far, no study had been done yet regarding the prevalence of glaucoma in Malaysia. It would be better if future studies emphasize the prevalence of glaucoma in Malaysia.

## 5. Conclusion

Overall glaucoma knowledge of our respondents was poor. Ethnicity, religion, education, occupation, and household income were associated with glaucoma awareness. Therefore, there is an urgent need to educate the public through all the possible media. Such action may increase awareness and detect the disease early and save costs for both patients and the government.

Therefore, there is a need for a comprehensive approach to raise awareness, for example, eye care professionals should be arranged to speak at public events about the importance of benefits of early glaucoma detection, and public figures and mass media should be involved in promoting glaucoma awareness. Glaucoma leaflets can be distributed at health clinics, social events, employee meetings, or after faith services. Also, NGOs and the private sector should be involved in promoting awareness of glaucoma among the public. A glaucoma awareness week can be established in Malaysia every year, the month of March to spread knowledge among the public.

## Figures and Tables

**Table 1:** Sociodemographic details of the study participants (*N* = 329).

Variable	Items	*N* (%)
Gender	Male	195 (59.3)
	Female	134 (40.7)

Race	Malay	236 (71.7)
	Chinese	3 (0.9)
	Indian	39 (11.9)
	Others	51 (15.5)

Education level	Secondary	215 (65.3)
	Tertiary	69 (21)
	Primary	30 (9.1)
	No formal education	15 (4.6)

Occupation	Working	230 (69.9)
	Nonworking	99 (30.1)

Chronic illness	No	290 (88.1)
	Yes	39 (11.9)

Family members with glaucoma	No	75 (90.4)
	Yes	8 (9.6)

**Table 2:** Awareness of glaucoma in the Malaysian community (*N* = 329).

Variable	Items		*N* (%)
Heard of the term glaucoma		Yes	63 (19.1)
		No	266 (80.9)

Heard of the definition of glaucoma		Yes	50 (15.2)
		No	279 (84)

Awareness of glaucoma		Aware	83 (25.2)
		Not aware	246 (74.8)

Source of information about glaucoma	TV	Yes	37 (44.6)
		No	46 (55.4)
	Radio	Yes	10 (12)
		No	73 (88)
	Newspaper/magazine	Yes	31 (37.3)
		No	52 (62.7)
	Internet	Yes	12 (14.5)
		No	71 (85.5)
	Relatives/friends	Yes	24 (28.9)
		No	59 (71.1)
	Physician	Yes	9 (10.8)
		No	74 (89.2)

**Table 3:** Knowledge of glaucoma in the Malaysian community (*N* = 329).

Variable	Items	*N* (%)
Anyone can have glaucoma	Yes	28 (33.7)
	No	26 (31.3)
	Not sure	29 (34.9)

Glaucoma is treatable	Yes	59 (71.1)
	No	6 (7.2)
	Not sure	18 (21.7)

Glaucoma is the same as cataract	Yes	24 (28.9)
	No	33 (39.8)
	Not sure	26 (31.3)

An eye doctor's check-up is the only way of early identifying glaucoma	Yes	57 (68.7)
	No	9 (10.8)
	Not sure	17 (20.5)

Glaucoma be developed by contacting a person with glaucoma	Yes	3 (3.6)
	No	53 (63.9)
	Not sure	27 (32.5)

Glaucoma caused by reading books, newspapers, or using a computer for a long time	Yes	31 (37.3)
	No	18 (21.7)
	Not sure	34 (41.0)

Glaucoma runs in families	Yes	27 (32.5)
	No	26 (31.3)
	Not sure	30 (36.1)

Who is most likely to have glaucoma?	People younger than 40 years old	3 (3.6)
	People older than 40 years old	27 (32.5)
	People of all ages are at the same risk	31 (37.3)
	Do not know	22 (26.5)

Glaucoma has asymptomatic course	Yes	33 (39.8)
	No	18 (21.7)
	Not sure	32 (38.6)

In glaucoma, vision is affected in the early course	Yes	51 (61.4)
	No	12 (14.5)
	Not sure	20 (24.1)
Does glaucoma affect	The central vision	6 (7.2)
	The side vision	16 (19.3)
	Both the central and side vision	11 (13.3)
	Do not know	50 (60.2)

Does glaucoma develop slowly over time or strike people suddenly?	Slowly	56 (67.5)
	Suddenly	4 (4.8)
	Do not know	23 (27.7)

Glaucoma causes blindness	Yes	55 (66.3)
	No	7 (8.4)
	Not sure	21 (25.3)

**Table 4:** Factors associated with awareness of glaucoma in the Malaysian community (*N* = 329).

Variables	Level of awareness (*N* = 329)		*X* ^2^ value	*P* value	Odds ratio
	Aware	Not aware			
Ethnicity			21.535	<0.001	5.720
Malay	76 (91.6)	160 (65.0)			
Non-Malay	7 (8.4)	86 (26.8)			

Religion			15.874	<0.001	5.836
Muslim	78 (94.0)	180 (73.2)			
Non-Muslim	5 (6.0)	66 (26.8)			

Educational level			26.766	<0.001	4.180
Tertiary education	34 (41.0)	35 (14.2)			
Below tertiary education	49 (59.0)	211 (85.8)			

Gender			3.455	0.063	—
Male	42 (50.6)	153 (62.2)			
Female	41 (49.4)	93 (37.8)			

Working status			0.0001	0.995	—
Working	58 (69.9)	172 (69.9)			
Not working	25 (30.1)	74 (30.1)			

**Table 5:** Factors associated with awareness of glaucoma in the Malaysian community (*N* = 329).

Variable	Mean (SD)	95% CI	*T* value	*P* value
Household income				
Aware	2495.00 (1209.98)	−627.79, −653.24	−2.655	0.008
Not aware	2000.13 (1018.87)			

Age				
Aware	35.39 (9.26)	−3.034, 1.848	−0.480	0.632
Not aware	34.79 (11.04)			

Types of awareness				
Aware by the term	26.27 (6.78)	−5.53, −0.05	−2.041	0.046
Aware by definition	29.06 (4.86)			

## Data Availability

The data that support the findings of this study are available from the corresponding author upon reasonable request.
